# Semisupervised Generative Autoencoder for Single-Cell Data

**DOI:** 10.1089/cmb.2019.0337

**Published:** 2020-08-04

**Authors:** Trung Ngo Trong, Juha Mehtonen, Gerardo González, Roger Kramer, Ville Hautamäki, Merja Heinäniemi

**Affiliations:** ^1^ University of Eastern Finland, School of Computing, Joensuu, Finland.; ^2^ University of Eastern Finland, School of Medicine, Kuopio, Finland.

**Keywords:** autoencoder, deep learning, generative, protein, semisupervised, single-cell, variational

## Abstract

**Single-cell transcriptomics offers a tool to study the diversity of cell phenotypes through snapshots of the abundance of mRNA in individual cells. Often there is additional information available besides the single-cell gene expression counts, such as bulk transcriptome data from the same tissue, or quantification of surface protein levels from the same cells. In this study, we propose models based on the Bayesian deep learning approach, where protein quantification, available as CITE-seq counts, from the same cells is used to constrain the learning process, thus forming a SemI-SUpervised generative Autoencoder (SISUA) model. The generative model is based on the deep variational autoencoder (VAE) neural network architecture.**

## 1. Introduction

*Single-cell RNA sequencing* (scRNA-seq) (Tang et al., 2009; Hedlund and Deng, 2018; Hwang et al., 2018) is a powerful tool to analyze cell states based on their gene expression profile with high resolution. RNA sequencing at single-cell level facilitates uncovering heterogeneous gene expression patterns in seemingly homogeneous cell populations. However, the current methods for gene expression profiling at single-cell resolution are prone to experimental errors, in particular, inefficient capture of mRNAs (Hwang et al., 2018). This capture inefficiency results in a general underestimation of the counts (dropout effect), which represents a major problem for single-cell analysis pipelines that rely on the mRNA counts.

Generally, the solution to the dropout problem has been posed as an *imputation* task, where missing counts are filled with estimated counts. The most recent approach is to model the dropout effect using the *zero-inflated* (ZI) model (Lambert, 1992), where a two-component mixture distribution is constructed, such that the first component models the dropout effect and the second component the observed counts. The effect of *overdispersion* is strongly presented in the scRNA-seq counts and the *negative binomial* (NB) distribution is seen as an appropriate fit to the observed data (King, 1989). Shallow imputation models that are based on ZINB or ZI log-normal models have been applied to single-cell data (Pierson and Yau, 2015; Risso et al., 2018). However, these models hypothesize a linear relationship between the latent space and the model parameters, which is quite a strong assumption (Lopez et al., 2018). To overcome the limitations of the linear models, deep neural network architectures have been proposed to resolve missing data (dropouts) (Eraslan et al., 2019).

An approach to this problem is to assume that there is a *latent* code that characterizes the cell type (or, more generally, cell state). Conditioning the ZINB distribution with these latent codes would allow sampling accurate transcriptome profiles. This approach was proposed by models such as single-cell variational inference (scVI) (Lopez et al., 2018) and single-cell variational autoencoder (scVAE) (Grønbech et al., 2018). In these techniques and the present article, the goal is to infer the posterior distribution of the latent code (Kingma and Welling, 2014). However, the sparseness of scRNA-seq data caused by low mRNA capture efficiency affects the quality of the estimated latent codes. To assess the quality of latent space representations of cell state, manual cell-type labeling of the obtained clusters based on marker gene expression has been used.

Before transcriptome profiling, analysis of surface protein markers has been the mainstream method to decipher cellular identity at single-cell resolution. Recently, Stoeckius et al. (2017) introduced the CITE-seq method that can combine scRNA-seq with such protein marker characterization from the same cells, thus providing complementary data on cell identity. Despite being limited to a small subset of expressed genes, the protein marker count data have the benefit that dropouts are rare. We believed these data could prove useful in assessing the quality of the latent representation. Moreover, it could be incorporated into model training to improve the single-cell model from scRNA-seq (Kingma et al., 2014). For the SemI-SUpervised generative Autoencoder (SISUA)^*^ model presented, we add the protein counts as an additional supervision signal (biological augmentation) with the goal of obtaining higher quality imputed counts and latent codes.

## 2. Methods

The task of unsupervised learning is to discover from the observed data (Bishop, 2006) hidden structure. In the case of scRNA-seq data, we assume that the true data manifold is of much lower dimension than the *embedded dimensionality* of the data. A single *batch* of cells has a total of *N* cells and each observation *x_j_*_, *i*_ is a non-negative integer, where *j* is the gene index. The representation of one cell in the estimated data manifold is typically denoted as a *latent* representation. We use this terminology in the following text.

### 2.1. Single-cell variational autoencoding

Autoencoders (Rumelhart et al., 1986) are deep neural network models that aim to learn the low-dimensional representation, based on a structure consisting of an *encoder* network, which performs the inference, a *bottleneck* layer, which constrains the dimensionality, and a *decoder* network, which performs the generation. The aim is to reconstruct the input signal with minimal loss, which is typically measured by the *mean squared error* function.

The limitations of deep autoencoders are highlighted in Higgins et al. (2016). The enoded vectors may not be continuous or allow easy interpolation which, coupled with the uncertainty in scRNA-seq data, could lead to excessive and meaningless variation in the latent space. As a result, the decoder will simply generate an irrelevant output under the slightest perturbation in input or latent space. Grønbech et al. (2018) and Lopez et al. (2018) suggest that a better latent representation could be learned using the variational method. We only observe **x**, but we would like to infer the characteristics of **z**, and hence, we compute the posterior

(1) $$p\left(z|x\right)=\frac{p\left(x|z\right)\cdot p\left(z\right)}{p\left(x\right)}.$$

The denominator is the marginal likelihood, which is intractable considering its involvement in all data points. Therefore, we approximate p(z|x) by another distribution q(z|x) (Kingma and Welling, 2014), and minimize the “distance” between the two distributions, which could give us a good approximation


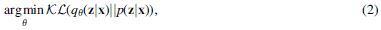


where *θ* is the neural network parameterization. We achieve the above minimization by maximizing the following (Kingma and Welling, 2014):


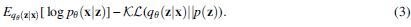


We choose the prior distribution 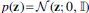, and using a deep neural network parameterized the conditional distribution $p_{\theta }\left(x|z\right)$.

### 2.2. Biological augmentation using semisupervised training (SISUA)

**FIG. 1.**
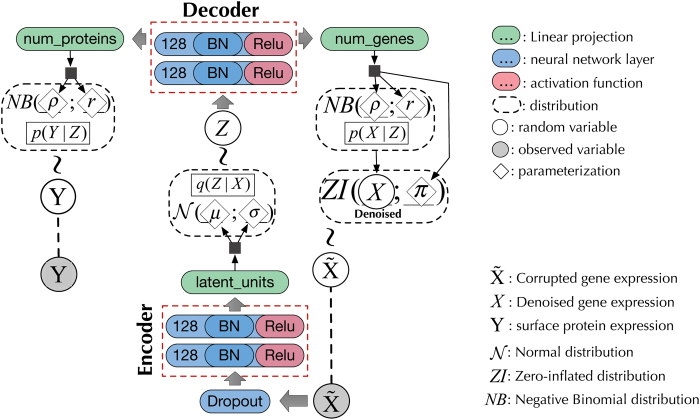
scVAE with the semisupervised extension. This is the design for both MOVAE and our proposed system SISUA. The implementation of ${ℒ}_{y}\left(y,f_{\theta }\left(y\right)\right)$ [Eq. (4)] is a major difference between SISUA and MOVAE. SISUA leverages probabilistic embedding to regulate the amount of information backpropagated from the supervised objectives, which is discussed in the Section 2.3. MOVAE, multioutput variational autoencoder; scVAE, single-cell variational autoencoder; SISUA, SemI-SUpervised generative Autoencoder.

In deep learning, it is possible to have more than one learning target and thereby models that learn a *shared* latent representation (i.e., *multi-task learning* Caruana, 1997). In our case, the protein labels are supplied for a subset of the input profiles, which are then modeled jointly to reconstruct mRNA and assign protein marker state, while the rest of the data are modeled only to reconstruct. The design of a multioutput variational autoencoder (MOVAE) is illustrated in Figure 1.

The label distribution of *Y* could be NB for count data (i.e., NB(Y|r,ρ)) or Bernoulli(Y|ρ) for probability data. The generative procedure of SISUA is as follows:


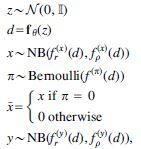


where $f_{r}^{\left(x\right)},f_{\rho }^{\left(x\right)}$ and $f_{r}^{\left(y\right)},f_{\rho }^{\left(y\right)}$ are linear projection function projecting the decoder vector into the corresponding dimension for *x* (denoised single-cell gene expression) and *y* (label data such as surface protein expression). *π* is the ZI rate modeled by Bernoulli variables.

We propose two design principles for semisupervised architectures that improve single-cell gene expression modeling:

- The use of labeled data should only be implicit, that is, no labeled data should be given as input during the evaluation process. The expense of labeling will typically preclude exhaustively labeled data. As a result, an algorithm, which implicitly encapsulates meaningful patterns from multimodal data into its latent space, would be more robust and practical.
- Unlike conventional semisupervised learning where an unsupervised objective is created to improve the supervised task (Kingma et al., 2014), semisupervised learning for single-cell data aims for the opposite. Since multiple losses have been known to compete with each other and hinder the major objective of the system (Goodfellow et al., 2014), Equation (4) is suggested when incorporating multiple losses into semisupervised systems.

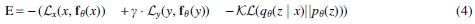


where *γ* is a hyperparameter representing the importance of the supervised tasks. Different *γ* are tested and fine-tuned in Section 5. ${ℒ}_{x}$ and ${ℒ}_{y}$ are likelihood functions for the corresponding distributions of the unsupervised and supervised variables.

**FIG. 2.**
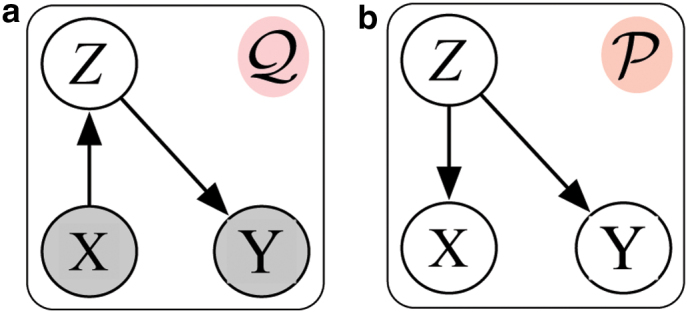
The design of the semisupervised systems and their probabilistic graphical models. The graph **(a)**, Q, is the inference model and the graph **(b)**, P, is the generative model.

Figure 2 illustrates the probabilistic graphical model of SISUA, which satisfies the above design principles. The inference process (Fig. 2a) parallels the biological relationship between mRNA and protein synthesis. The generative process (Fig. 2b) enables the sampling of both gene expression and protein marker levels from a biologically motivated latent space.

### 2.3. Probabilistic embedding for biological data

**FIG. 3.**
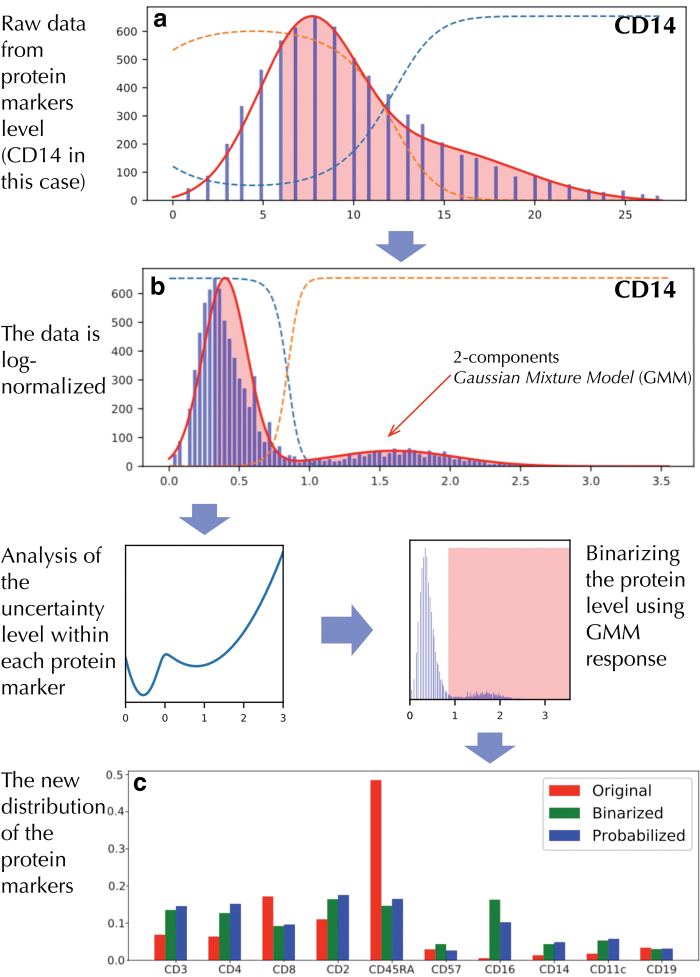
The process of probabilistic protein embedding using data for protein *CD14* as an example. (**a**) and (**b**) illustrate a 2-component GMM filled on raw count and log-normalized data. The comparison between the distribution of protein markers before and after the process is shown at the bottom figure **(c)**, which shows that the binarized and probabilized values are more balanced than the original distribution.

Inspired by Reynolds (2009), we propose a generalized approach for incorporating multimodal biological data into the unsupervised algorithm. A Gaussian mixture model (GMM) is used to represent general, cell-independent feature characteristics. In our case, the GMM is used to capture different modes of protein activation based on the surface protein levels. Two considerations motivate the application of the GMM in biological data:

- The data often come from sources (e.g., different measurements) with different characteristic scales and technical variability.
- The distribution of the data is often skewed and imbalanced. For example, Figure 3c indicates abnormally high abundance of “*CD45RA*.” This could trigger the false perception that “everything is *CD45RA*” during the optimization of the deep neural network (Dalyac et al., 2014; Hensman and Masko, 2015).

For each protein, a two-component GMM is trained,


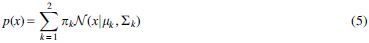


where *x* is a single-dimension protein level. *x* could be the raw value in Figure 3a or the log-normalized value in Figure 3b. *μ_k_* and Σ_*k*_ are the mean and covariance vectors of the Gaussians, and *π_k_* is the mixture weight. Those parameters are the maximum likelihood estimates (Reynolds, 2009).

We utilize a set of GMMs associated with each protein. These GMMs are used to generate the response of the protein to each individual cell, “probability” protein expression. They can be thresholded, as in Figure 3, to yield binary variables indicating the presence or absence of protein in each cell.

## 3. Experimental Setups

### 3.1. Data sets

The experiments were run on two data sets. The first data set, peripheral blood mononuclear cells (PBMC), consists of 12,039 human peripheral blood mononuclear cells, generated using the 10x Genomics platform. This data set includes cell-type labels assigned by manual examination of clusters (Zheng et al., 2017). The second data set, peripheral blood CITE-seq data, was downloaded from 10x Genomics^†^. Protein marker levels were available for a total of 14 specific antibodies and 3 control (IgG) antibodies. Here, we utilized the subset of lymphoid cell populations (Ly) (4697 cells, 2000 most variable genes). In addition to raw counts for mRNA, centered log-ratio-normalized (CLR-normalized) antibody derived tag (ADT) counts were used for model evaluation and training.

To evaluate the generalizability, we split each data set into disjoint training (90%) and testing 10% subsets. For imputation benchmarking, we measure the robustness of the algorithm by corrupting the original training data and then using the learned algorithm to provide denoised gene expression. Binomial data corruption was applied as in Lopez et al. (2018). Twenty-five percent of the matrix entries are randomly selected and replaced with a Bin(n,0,2) random variable, where *n* is the original count of the given entry.

### 3.2. Learning algorithms

Three state-of-the-art unsupervised baselines were selected for comparison with MOVAE and SISUA:

- Deep count autoencoder (DCA) is a denoising autoencoder that takes the count distribution (Eraslan et al., 2019).
- scVI is a framework using deep probabilistic inference to model observed expression values, accounting for the technical variability of the measurements (Lopez et al., 2018).
- scVAE (Grønbech et al., 2018) also utilizes deep generative modeling. This approach was arguably the least sophisticated because the cell size (or library size) is implicitly modeled via parameterization of NB distribution.

**FIG. 4.**
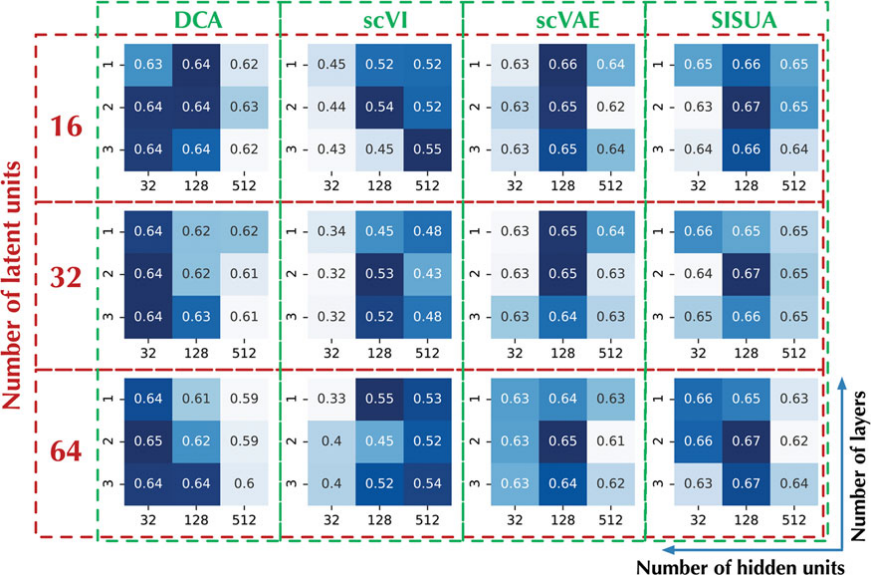
Tuning the deep neural network architecture using grid search. We varied the number of latent units (16, 32, 64), together with three different configurations for the number of hidden layers (1, 2, 3) and the number of hidden units (32, 128, 512). For evaluation, we use combined correlation scores of Pearson and Spearman's correlation calculated using all marker gene and protein pairs on the test set. Darker blue color represents better performance.

We configured all frameworks, in terms of number of hidden layers, hidden units, and latent units, similar to the design as in Figure 1. In addition, the same optimization algorithm (ADAM; Kingma and Ba, 2015) and training parameters were used for all models. The choice of these hyperparameters is the result of a tuning process illustrated in Figure 4. It indicates a design balancing among the number of hidden layers (i.e., 2 layers), and the number of units (128 hidden and 32 latent units) could give the best results for most of the models. Notably, the color pattern is matching between scVAE and SISUA; hence, introducing semisupervised learning does not significantly alternate the tuned hyperparameters. As a result, semisupervised extension could be rapidly incorporated into existing models.

## 4. Experiments and Results

In the following experiments, we focused on three major functions of an autoencoding model:

- The output space, the imputed gene expression profile, is evaluated using (1) per-cell marker protein levels (PBMC CITE-seq), or (2) per-cell assigned labels from the manual examination of data (PBMC RNA-seq).
- The latent space, as a low-dimensional representation of the data, is evaluated for biological tasks.
- The semisupervised space, expressing the supervised labels, is a unique feature of SISUA. We evaluate the soundness of this space by its connection to the output and latent space and utilizing the ground truth labels.

### 4.1. Correlation of marker mRNA gene expression and surface protein levels

**FIG. 5.**
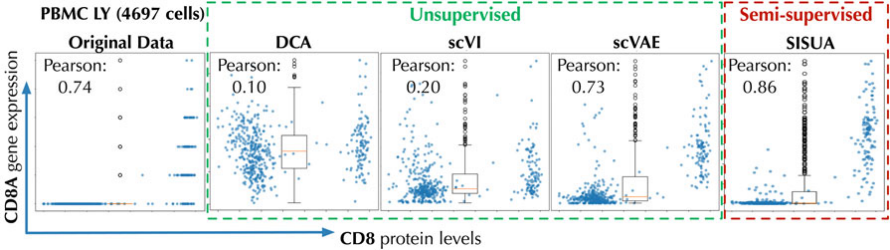
Correlation between *CD8A* gene mRNA count data and protein surface marker levels in PBMC lymphoid cells. Semisupervised models are highlighted by red dashed box.

Because assaying marker protein levels is less prone (for technical reasons) to the dropout issues that plague mRNA levels for the corresponding genes, cell surface marker protein expression can be used as “ground truth” for evaluating known cell states and cell types. Thus, the denoised corresponding mRNA levels for the same markers can be evaluated in an unbiased manner (Stoeckius et al., 2017; Eraslan et al., 2019) (Fig. 5).

**FIG. 6.**
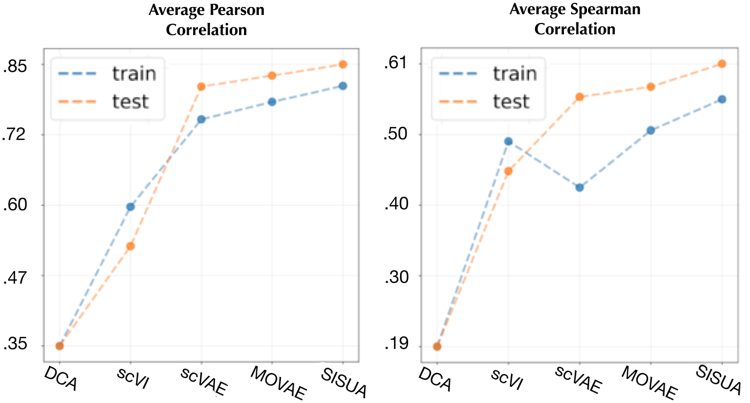
The average Pearson and Spearman's correlation for all marker gene/protein pairs.

As exemplified by the T cell marker *CD8,* nonzero counts are observed when protein levels are high. This correlation is poorly modeled by DCA and scVI that impute counts also to cells with low surface protein levels, while scVAE preserves the correlation. Semisupervised learning consistently improved the correlation across all marker gene and protein levels (Fig. 6). In all cases, SISUA was able to restore missing gene expression that is biologically plausible.

### 4.2. Structural integrity of denoised space

The experiment evaluates whether the imputed gene expression spaces still maintain the same essential variability model as the original data. To confirm that relevant biological information was preserved during the denoising processing, we inspected two properties:

- First, by coloring the cell types (first row) we could confirm whether similar cells still form clusters that reside in the same position in the total variability space.
- Second, the cell size (colored in second row) was compared with that in the original data.

**FIG. 7.**
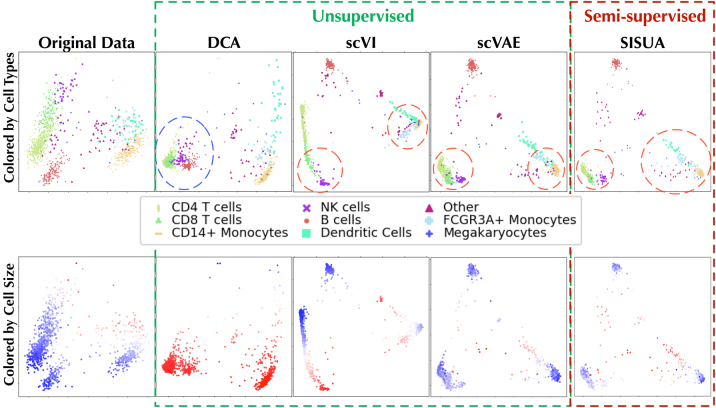
A PCA model trained using original gene expression (the data before corruption) of the PBMC 10x data set was used to project the denoised gene expression from different models into its space. The top row is colored by cell type, and the bottom row by denoised cell size (red color indicates large, white color midrange, and blue small cell size).

In Figure 7, we observed that DCA is altering both the cell types and cell size (blue circle), yet keeps a high amount of variability. scVI has very low variability, but performs good on cell-type clustering and preserving cell sizes. scVAE provides better variability but slightly worse cell size. SISUA improves the variability compared with scVAE and also improved the cell-type model (red circles). In addition, SISUA slightly increased the cell size compared with scVAE although this was not explicitly modeled.

### 4.3. Separation of cell types in latent space

**FIG. 8.**
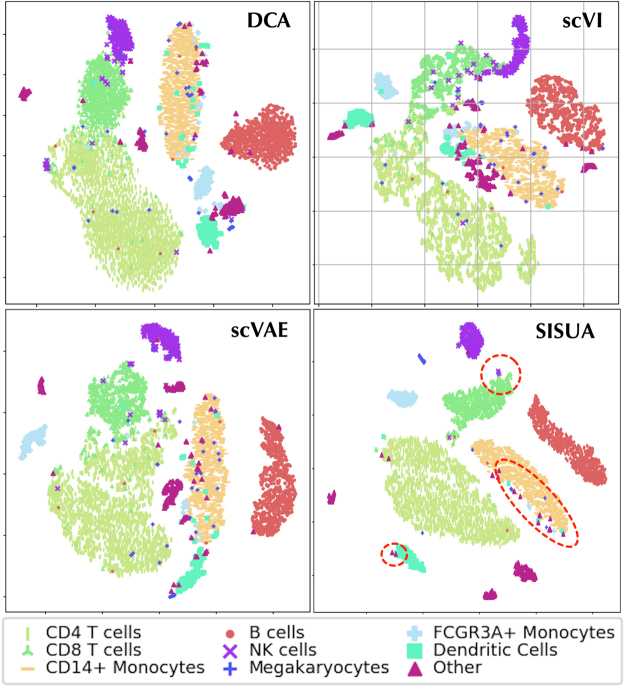
t-SNE visualization of the latent space for PBMC 10x data set, and binary cell-type labels are used for coloring.

**FIG. 9.**
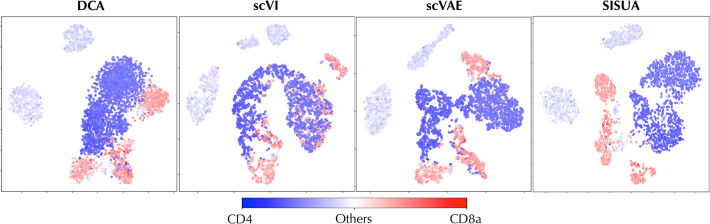
t-SNE visualization of the latent space for PBMC Ly data set, and the activation levels of protein *CD8a* (dark red tones) and *CD4* (dark blue tones) are shown as heatmap.

A biologically informative latent space should have a clear separation between different cell types. To evaluate this, the points on the t-SNE maps are colored by their “ground truth” labels (manual labels in Fig. 8 and protein marker state in Fig. 9).

In Figure 8, many outliers are present in the DCA, scVI, and scVAE results. scVI shows extra confusion among cells by grouping many clusters close together. Notably, most of the outliers were classified as “Other” cells. SISUA yields a cleaner cluster structure with fewer outliers. Moreover, there is a subtle group of “*NK* cells” placed adjacent to the “*CD8 T* cells” (SISUA figure panel). This *rightly* calls into question the mutually exclusive labeling of cells because there, in fact, exist “*NK* cells,” which are also “T cells.” Immunologists recognize an entity called *NKT* cells as a separate cell type. Among the models, only the latent space representation of SISUA clearly indicates the prominent characteristics of the “T cells” within this small group of “*NK* cells.” Notably, this similarity is learned without extra information about the “T cells.”

In PBMC Ly, each cell is characterized by multiple protein levels, which include markers for similar cell types (such as *CD4*- or *CD8*-positive T cells) that often get tangled up in the latent representation of mRNA data. Compared with all unsupervised representations, SISUA achieves a strong separation between “*CD8*” protein and “*CD4*” protein in its latent space (Fig. 9). This division is biologically plausible and the algorithm is able to learn this pattern independently without explicit indicators.

**FIG. 10.**
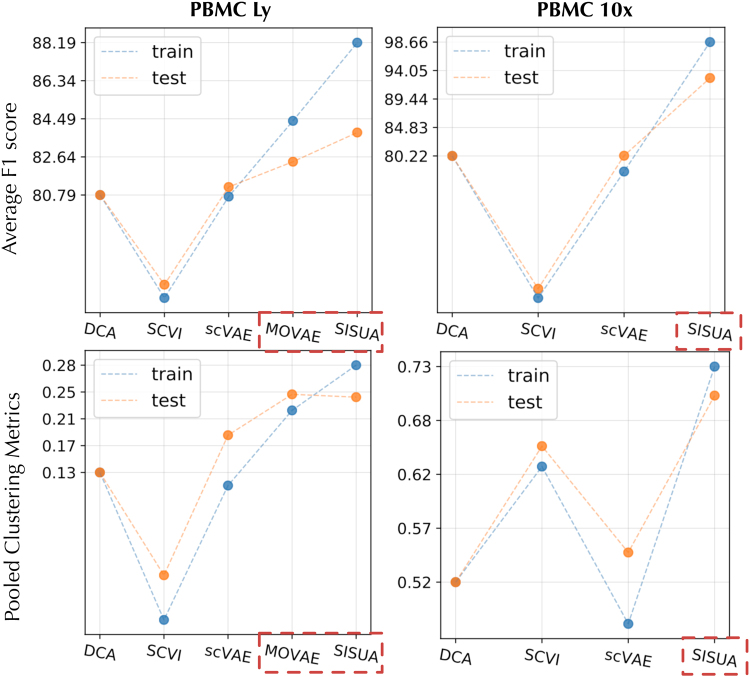
Latent spaces are evaluated by two benchmarks. First, the average of F1 scores from a streamline protein/cell-type classifiers; Second, the pooled clustering metric calculated by averaging four measurements: *ARI*—adjusted rand index, *ASW*—silhouette score, *NMI*—normalized mutual information and *UCA*—unsupervised clustering accuracy. The results are reported for PBMC Ly and PBMC 10x data sets. The train results are shown in blue dots, and the test results in orange dot. Semisupervised models are highlighted by red dashed boxes.

In addition, we evaluate the learned latent spaces quantitatively using two different approaches. First, we feed the learned latent to a secondary classifier that is trained to classify protein markers or cell labels. The results are shown in Figure 10. We notice that in terms of the classification F1-score, SISUA is clearly the best model. The testing performance of the semisupervised models is degraded when compared with the training portion, but still clearly win over the fully unsupervised variants. As a second method, we pool the external validity indices used to assess clustering quality; these results are shown in the second row of Figure 10. The results indicate that the information encapsulated in the semisupervised latent space is higher than the other models.

### 4.4. Predictive protein distribution

**FIG. 11.**
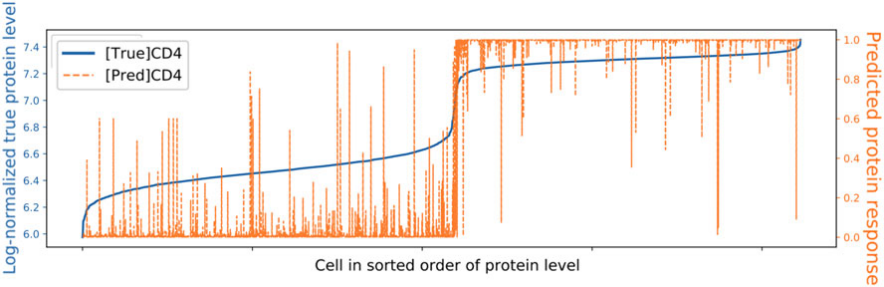
Comparing the original protein level and predicted protein response on PBMC Ly data set.

Figure 11 illustrates how the SISUA model has learned to predict protein marker levels, visible as a high correlation of the predicted level and the “ground truth” protein expression. However, there are many unexpected peaks mismatching with the “ground truth.”

**FIG. 12.**
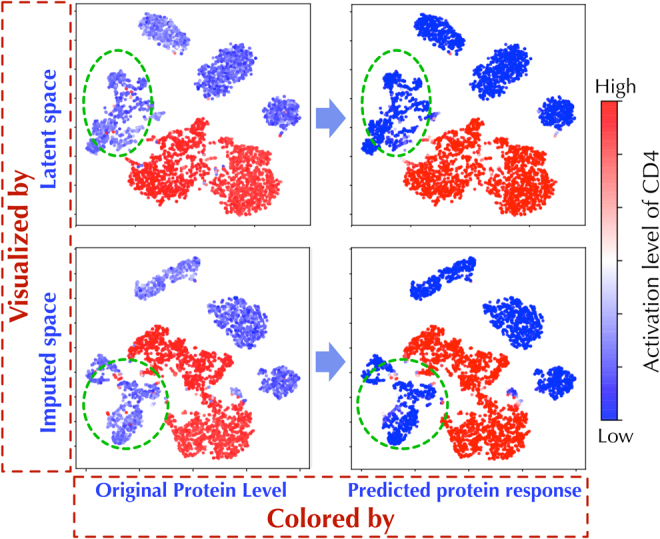
Both latent space and imputed space are transformed into 2D scatter visualization using t-SNE. The two figures in the left column are colored by the “ground truth” protein and the right column is colored by the predicted protein level.

In Figure 12, the model learns to calibrate many faulty points in the given protein labels itself. The figures colored by the predicted protein level indicate a more relevant structure, where many outliers for “*CD4*” protein levels are cleaned and grouped into neat clusters (highlighted by green circles). Notably, these are observed in both latent and denoised spaces, and hence, SISUA has been able to capture relevant biological connections at multiple levels.

## 5. Efficiency and Scalability

In this section, we focus on two different aspects of efficiency for semisupervised learning:

- Quantitative: the amount of supervised data that has to be gathered until significant improvement.
- Qualitative: the contribution of the supervised objective for the overall learning process.

Consequently, we evaluate the scalability of semisupervised training to large data sets. The experiments assess the potential of deploying SISUA for real-world application.

### 5.1. Quantitative efficiency

**FIG. 13.**
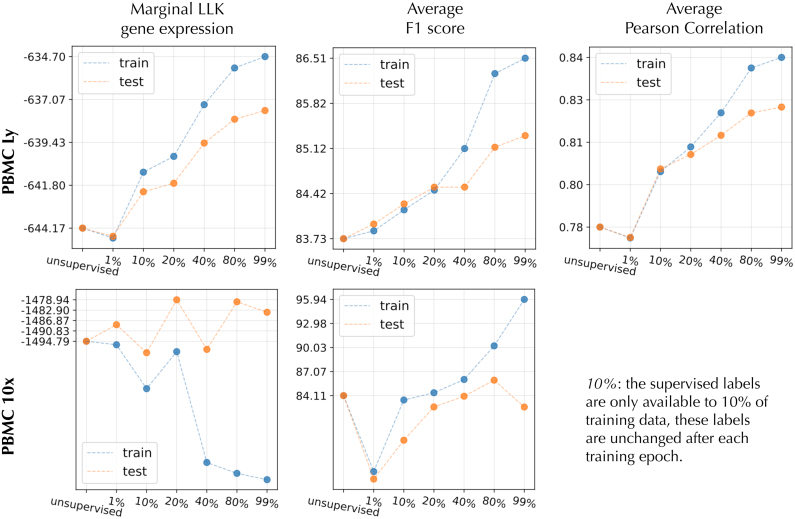
The performance on two data sets (PBMC Ly and PBMC 10x) is shown for different amount of labels utilized in training. The *X*-axis represents seven systems with an increasing amount of labeled data available for the semisupervised objective (*Note:* no marker gene/protein pair is available for PBMC 10x). We measured three different metrics: the marginal log-likelihood, average F1 of protein/cell-type classifier in the latent space, and average correlation between marker gene and the protein.

In Figure 13, we noticed in the PBMC Ly subset that adding only 1% of labels degraded the performance in all cases, while the addition of 10% gave a clear boost in all three metrics. As expected, the addition of labeled examples systematically improved the model in the PBMC Ly. In the case of PBMC 10x, the situation is not as clear, since the average F1 improved until 80% of the training examples are labeled. However, the marginal log-likelihood does not show systematic behavior. One reason for this nonsystematic behavior could be errors in PBMC 10x binary cell-type labels.

### 5.2. Qualitative efficiency

**FIG. 14.**
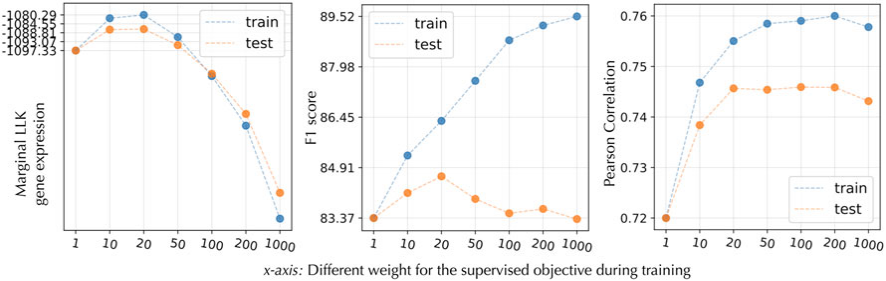
Performance of SISUA when *γ* (i.e., the weight of semisupervised objective) is varied.

Figure 14 emphasizes the important role of semisupervised learning. The algorithm must balance the benefit of supervised learning via *γ*. We notice in all cases that *γ* of more than 20 gives clear improvement over the unsupervised case. However, when *γ* is increased more, the model starts to favor more the proteins than gene expression in the reconstruction.

### 5.3. Scalability

**FIG. 15.**
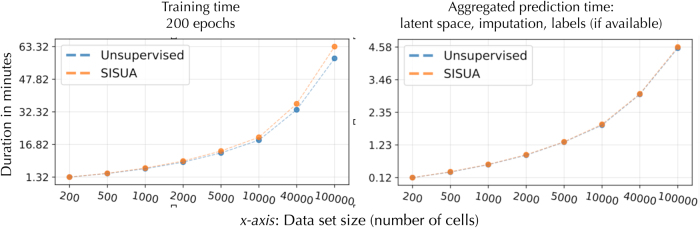
Running time for the training phase (left figure) and evaluation phase (right figure) for the unsupervised model and semisupervised model (SISUA). The algorithms were run on an eight-core Intel Xeon CPU E5-1630, and one NVIDIA GeForce GTX 1080.

Finally, the training algorithm running time as a function of number of cells is shown in Figure 15. We observed that the semisupervised extension added a very minor increment to the running time when compared with the unsupervised variant. It is at most 8% longer compared with the unsupervised for 100,000 cells. Notably, SISUA introduces no extra running time during the evaluation phase, since no extra data are needed. Instead, we get the extra benefit of obtaining protein-level predictions.

## 6. Conclusion

In this article, our task is to use weak supervision to improve unsupervised analysis of single-cell gene expression profiles. We design a new model, SISUA, which leverages a small amount of labeled data to produce more biologically meaningful latent representations. Our results support the merits of this semisupervised extension. In addition to more interpretable latent representations, the method improves imputation of mRNA sequence counts. SISUA is also capable of predicting cell types or surface protein levels from transcriptomic data, which extends its utility to diagnostic contexts. Finally, we propose general guidelines for implementing efficient and practical semisupervised systems that can leverage the variety of data types available for single-cell modeling.

## Notes

^*^ Code and reproducibility: https://github.com/trungnt13/sisua

^†^ pbmc_10k_protein_v3_filtered_feature_bc_matrix.tar.gz
